# Foliar zinc biofortification effects in *Lolium rigidum* and *Trifolium subterraneum* grown in cadmium-contaminated soil

**DOI:** 10.1371/journal.pone.0185395

**Published:** 2017-09-26

**Authors:** Maria J. Poblaciones, Paul Damon, Zed Rengel

**Affiliations:** 1 Department of Agronomy and Forest Environment Engineering, University of Extremadura, Badajoz, Spain; 2 School of Agriculture and Environment, The University of Western Australia, Perth, Australia; RMIT University, AUSTRALIA

## Abstract

Zinc (Zn) is an important micronutrient that can alleviate cadmium (Cd) toxicity to plants and limit Cd entry into the food chain. However, little is known about the Zn-Cd interactions in pasture plants. We characterized the effects of foliar Zn application and Cd uptake by ryegrass (*Lolium rigidum* L.) and clover (*Trifolium subterraneum* L.) grown on Cd-contaminated soils; all combinations of foliar Zn applications (0, 0.25 and 0.5% (w/v) ZnSO_4_·7H_2_O) and soil Cd concentrations (0, 2.5 and 5 mg Cd kg^-1^) were tested. For both plant species, soil concentrations of DTPA-extractable Cd and Zn increased with an increase in the Cd and Zn treatments, respectively. Compared with *L*. *rigidum*, *T*. *subterraneum* accumulated, respectively, 3.3- and 4.1-fold more Cd in the 2.5-Cd and 5-Cd treatments and about 1.3-, 2.3- and 2.8-fold more Zn in the No-Zn, 0.25-Zn and 0.5-Zn treatments. Also, DTPA-Zn concentration was higher in soil after *T*. *subterraneum* than *L*. *rigidum* growth regardless of Zn applications. Foliar application of 0.25% (w/v) Zn significantly decreased the total Cd concentration in shoots of both species grown in the Cd-contaminated soil and ameliorated the adverse effects of Cd exposure on root growth, particularly in *T*. *subterraneum*.

## Introduction

At least two billion people worldwide are affected by zinc (Zn) deficiency, which is one of the most common micronutrient malnutrition problems [1–2]. Zinc is an essential nutrient in plants, animals and humans, and its entry into the food chain is directly related to the chemical solubility of Zn in soils [1]. In some areas of Western Australia with low Zn solubility in soils due to high pH, low organic matter and/or low soil moisture, Zn deficiency has been found in plants [3], cattle and humans [4]. In livestock and humans, low Zn intake has been associated with severe health complications, including impairments of growth and the immune system combined with increased risk of infections, DNA damage and cancer development [5].

In plants, Zn deficiency can cause serious crop production problems because Zn is required for photosynthesis, protein synthesis, detoxification of toxic oxygen free radicals, pollination, growth regulation and disease defence mechanisms [6]. In crops grown on soils with a range of Zn availabilities, foliar Zn application (typically 0.25 to 0.6% (w/v) zinc sulphate) has been shown as an effective technique of agronomic biofortification to increase significantly Zn concentration in many crops [2–3,7–8]. Penetration of Zn-containing foliarly-applied solution would occur partially through the cuticle and mostly through the stomata [9].

An important benefit of Zn is the alleviation of cadmium (Cd) toxicity in plants, animals and humans [10]. This antagonist effect has been reported in the 1960s by Perkins [11] and Hawf and Schmid [12]. Later studies showed that this interaction can also be synergistic or non-existent, depending on plant species, growth conditions, and Cd and Zn status of soils and plants [13–14]. Cadmium, occupying the 67^th^ position in abundance among the 90 naturally occurring elements on earth, has adverse effects on soil biological activity and is highly toxic to living organisms [15]. It is ranked as the 7^th^ toxicant in the Priority List of Hazardous Substances by the Agency for Toxic Substances and Disease Registry [16–17]. In plants, Cd toxicity results in inhibition of germination, decreased seedling vigour and shoot and root growth, decreased root hydraulic conductivity [15], chlorophyll content and stomata opening and conductance, and interference with absorption and translocation of nutrients, mainly Ca, Fe and Mg [18]. In animals and humans, Cd toxicity is associated with renal, neurological, skeletal and other toxic effects, including reproductive toxicity, genotoxicity and carcinogenicity [19]. Given that feed and food are the main routes of Cd intake in animals and humans [18], minimising the entry of Cd into the food chain is becoming a focal point for the relevant regulatory and other authorities.

Cadmium accumulates in soils mainly from anthropogenic sources such as phosphate fertilizers and sewage sludge, but also by emissions from power stations, metal and cement industries, and vehicles [20–21]. In Australian agricultural soils, increasing concentration of Cd is mainly due to an application of phosphate fertilisers and sewage sludge [22].

Soil Zn application is a relatively inexpensive, quick and effective approach to avoid Cd contamination of food [23]. Zinc can alleviate Cd-induced physiological damage to biomolecules and membranes [24] and decrease Cd uptake, minimize chlorophyll breakdown and lipid peroxidation, and repair DNA damage caused by Cd exposure [25–26]. The usefulness of soil applications of Zn (5–15 mg Zn kg^-1^ soil) in decreasing uptake, translocation and accumulation of Cd in plants is well documented [13, 27]. However, there is little information about the effect of foliar Zn application (implying lower doses of Zn than soil applications) on Cd accumulation; only Akay and Köleli [28] in barley (*Hordeum vulgare* L.) and Saifullah et al. [26] and Sarwar et al. [29] in wheat (*Triticum aestivum* L.) studied it previously. Moreover, no information could be found for pasture plants despite their importance in the animal health and Cd entry into the food chain. The aim of this study was to evaluate foliar Zn application on two pasture plants (grass *Lolium rigidum* L. and legume *Trifolium subteranneum* L.) grown in Cd-contaminated soils in terms of increasing Zn and minimising Cd shoot accumulation, and the effects on biomass production and quality (protein and phytate concentrations).

## Material and methods

### Preparation of contaminated soil

A Cambic Arenosol soil without detectable Cd was purchased from Rocla Quarries company, a commercial operator with an appropriate license to operate; the soil was collected from the area of Gingin in Western Australia (31°46′S, 115°86′E). The soil was air-dried and sieved to <5 mm. Four subsamples of the sieved soil were analysed for various physico-chemical properties. Soil texture (determined gravimetrically) was sandy. The soil had pH 6.4 (10 g soil:25 mL deionised H_2_O), electrical conductivity 0.031 dS m^−1^, organic carbon 2.9 g kg^-1^ (Walkley-Black method), available phosphorus 18 mg kg^−1^ and potassium <15 mg kg^−1^ (Colwell method), soil nitrate nitrogen 1 mg kg^−1^ and ammonium nitrogen 1.4 mg kg^−1^ [extracted with 1 M potassium chloride solution for 1 hour at 25°C and measured on a Lachat Flow Injection Analyzer (Ames, Iowa, USA)]. Extractable Cd and Zn in soil were <0.002 (i.e. below detection limit) and 0.3 mg kg^-1^, respectively, determined according to Lindsay and Norvell [30] by extraction with DTPA (diethylenetriamine pentaacetic acid) using a soil:solution ratio of 1:2 and shaking time of 2 hours. Extracted Cd and Zn were determined by inductively-coupled plasma optical emission spectroscopy (ICP-OES, Vista-Pro Axial, Varian Pty Ltd, Mulgrave, Australia). The certified soil reference material and blanks were included in each batch of samples. All the results were reported on a dry weight basis.

Cadmium (as CdCl_2_×2·1/2 H_2_O, analytical grade, Ajax Chemicals, Sydney, Australia) was dissolved in Milli-Q water and added to soil at concentrations of 0, 2.5 or 5 mg Cd kg^−1^. To ensure Zn was the only nutrient limiting growth, the following basal nutrients (in mg kg^-1^) were added to soil as solutions: 90.2 KH_2_PO_4_; 139.9 K_2_SO_4_; 40.1 MgSO_4_·7H_2_O; 95.2 NH_4_NO_3_; 150.3 CaCl_2_·2H_2_O; 10.0 MnSO_4_·H_2_O; 2 CuSO_4_·5H_2_O; 0.5 CoSO_4_·7H_2_O; 0.2 Na_2_MoO_4_·2H_2_O, and 0.7 H_3_BO_3_. The amended soils were placed in plastic bags and equilibrated in a dark room at 25°C for 10 days. The soil samples were collected after equilibration and analysed for soil pH and extractable Cd.

### Experimental setup

The experiment was arranged in a factorial pattern with four replications. The first factor was the soil Cd concentrations resulting from the addition of 0, 2.5 and 5 mg Cd kg^-1^ (No-Cd, 2.5-Cd and 5-Cd, respectively). The second factor was the foliar application of Zn (7.5 mL pot^-1^), with distilled-water spray (No-Zn), 0.25% or 0.5% (w/v) of ZnSO_4_·7H_2_O (0.25-Zn or 0.5-Zn) onto 4-week-old plants in the late afternoon; the volume of sprayed solution was sufficient to wet all the leaves. The pot surface was covered with polythene at the base of the plants to prevent contact between the solution and soil. The final number of pots was 72.

Twenty seeds of *Lolium rigidum* cv. Wimmera or *Trifolium subterraneum* cv. Seaton Park were sown in each 3.3-L pot (140 mm diameter at the top and 140 mm in height) containing 2.6 kg of soil. Seeds were surface-sterilised by soaking in 80% v/v ethanol for 60 s and washing thoroughly with sterile water. Two weeks after emergence, the seedlings were thinned to ten plants per pot. Soil moisture content was maintained around 60% of the water holding capacity by watering the plants every 2 days with deionised water. In order to ensure soil aeration and prevent excessive salt accumulation and waterlogging, four drainage slits (2 cm wide) were made 2 cm above the bag base. During plant growth, nitrogen (33.3 mg kg^-1^ as NH_4_NO_3_ solution) was applied every 14 days. There was no incidence of pest or diseases during the experiment.

Plants grew between 4^th^ May and 29^th^ June 2015 in a naturally-lit glasshouse located at The University of Western Australia in Perth (31°57′ S, 115°47′ E), Australia. During the experiment, the average temperature in the glasshouse was 23 ± 4°C during the day and 17± 4°C during the night. Light intensity varied between 250 and 1100 μmol photon m^-2^ s^-1^, and relative humidity varied from 40 (midday) to 85% (midnight).

### Plant analysis

The plants were harvested after 8 weeks of growth. Soil samples were collected to analyse the extractable Cd and Zn at the end of the experiment as explained above. Shoots were cut just above the soil surface, and their base was washed with deionised water; roots were separated from soil by washing with running deionised water over a mesh and rinsing with deionised water three times. Subsamples of fresh roots were used to determine root length and average diameter. Roots were spread out on a Perspex tray to avoid overlapping, and an image of the roots was captured using a flat-bed scanner (EPSON Perfection V700/V750, operating resolution 400 dpi) and WinRhizo 5.0 ATM software (Regent Instruments Inc., Montreal, QC, Canada). Shoots and the remaining root subsamples were air-dried at 60°C for 72 hours in a forced-air cabinet until constant weight, and weighed.

Shoot nitrogen content was determined using the Dumas combustion method and a Leco FP-428 analyser (Leco Corporation, St. Joseph, MI, USA). Grain protein was determined by multiplying total N by 6.25 as a conversion factor. Total shoot Zn and Cd concentrations were determined by ICP-OES (as described above) after digesting plant material in a heated mixture of concentrated nitric and perchloric acids [8].

Given that not only total shoot (forage) Zn concentration is important, but also its bioavailability, we determined phytate concentration in shoots as well. The phytic acid assay was based on precipitation of ferric phytate and measurement of iron (Fe) remaining in the supernatant [31]. Phytic acid was extracted from about 0.2 g of ground shoots in 10 mL of 0.2 M HCl (pH 0.3) after shaking for 2 h. One mL of supernatant was treated with 2 mL of ferric solution [NH_4_Fe(SO_4_)_2_·12 H_2_O] in a boiling water bath for 30 min. After cooling, samples were centrifuged, and 1 mL of supernatant was treated with 1.5 mL of 0.064 M bipyridine to measure Fe. After mixing, the solution was incubated for 10 min at room temperature, and the light absorbance was measured with a spectrophotometer at 419 nm. The molar ratio between phytate and Zn was calculated.

### Statistical analyses

For each plant species, data were subjected to two-way ANOVA, including the main effects (soil Cd additions and foliar Zn application) as well as their interaction in the model (soil Cd, foliar Zn, soil Cd*foliar Zn). When significant differences were found in ANOVA, means were compared using Fisher’s protected least significant difference (LSD) test at *P* ≤ 0.05. All analyses were performed using *Statistix v*. *8*.*10 for Windows (Analytical Software*, *Tallahassee*, *FL*, *USA)*.

## Results and discussion

### DTPA-extractable Cd and Zn in soil

The initial soil analyses before the experiment showed that DTPA-extractable Zn was 0.3 ± 0.02 mg Zn kg^-1^ and DTPA-extractable Cd (after equilibration of different soil Cd treatments) 0.01 ± 0.01, 1.83 ± 0.08 and 4.23 ± 0.25 mg Cd kg^-1^ in the No-Cd, 2.5-Cd and 5-Cd treatments, respectively. The initial Zn value was lower than the widely accepted critical Zn concentration of 0.5 mg Zn kg^-1^ [32]. As it will be discussed further down, even though there may have been some root and shoot growth limitation caused by low Zn availability in the No-Zn treatment, it was relatively small; in contrast, shoot Zn concentration in both plant species was above the critical level in the No-Zn treatment, suggesting that Zn supply in the No-Zn treatment was not particularly growth limiting. After harvesting plants, DTPA-extractable soil Cd was significantly influenced by both treatments (soil Cd and foliar Zn), but the interaction was significant only in case of *L*. *rigidum* (Table 1).

**Table 1:** Values for statistic F from 2-way ANOVA for the parameters measured in the treatments with *L*. *rigidum* and *T*. *subterraneum*.

	*L*. *rigidum*			*T*. *subterraneum*		
	Cd	Zn	Cd*Zn	Cd	Zn	Cd*Zn
D.F.	2	2	4	2	2	4
Soil DTPA Cd	2557**	12***	5.9**	791***	3.9*	1.6
Soil DTPA Zn	1.8	5.5*	0.8	2.5	6.5*	1.0
Root dry weight	2.6	2.9	1.6	0.9	15***	3.6*
Root length	4.5*	6.0*	0.9	38***	16***	5.6**
Average root diameter	0.9	1.0	0.9	3.1	3.8*	1.5
Shoot dry weight	0.9	6.6**	1.5	6.1**	11***	2.4
Shoot Cd concentration	3971***	20***	6.3**	790***	2.0	3.8*
Shoot Zn concentration	2.5	42***	0.5	3.5	147***	1.2
Shoot phytate:Zn ratio	3.9*	135***	0.6	14***	309***	11***
Shoot protein concentration	1.2	0.2	0.2	1.1	5.9*	2.0

D.F. Degrees of freedom; Level of significance:

* P ≤ 0.05;

** P < 0.01;

*** P < 0.001

In the treatments with either plant species, DTPA-extractable soil Cd concentrations were similar and, as expected, increased with the increase in the soil-applied Cd (Tables 2 and 3). However, in the foliar treatment with 0.25% w/v ZnSO_4_·7H_2_O, DTPA-extractable soil Cd was lower after growth of either species, with a significant decline in case of *L*. *rigidum* in the 5-Cd treatment (Tables 2 and 4). This finding raises a possibility of using a low-dose foliar Zn (0.25% w/v ZnSO_4_·7H_2_O that is equivalent to a dose of 3 kg Zn ha^-1^ or 1.7 mg Zn kg^-1^) to decrease the availability of soil Cd; this Zn dose was lower than those applied to soil by Adiloglu [27] (15 mg Zn kg^-1^) or Köleli et al. [11] (10 mg Zn kg^-1^) that decreased Cd accumulation in plants and alleviated Cd toxicity symptoms in cereals. No prior report could be found on using foliarly-applied Zn to influence the availability of soil Cd.

**Table 2:** DTPA-extractable soil Cd and shoot Cd concentration in *L*. *rigidum*, and root DW, root length, shoot Cd concentration and phytate:Zn molar ratio in *T*. *subterraneum* as affected by the interaction between the soil Cd and Zn foliar treatments. Mean ± standard error.

Treatment	*L*. *rigidum*		*T*. *subterraneum*			
	DTPA-extractable soil Cd (mg kg^-1^)	Shoot Cd concentration (mg kg^-1^)	Root DW (g plant^-1^)	Root length (cm plant^-1^)	Shoot Cd concentration (mg kg^-1^)	Shoot phytate:Zn ratio
**No-Cd**						
No-Zn	<d.l.* e	<d.l.** e	0.10 ± 0.01 c	478 ± 34 bc	<d.l.** d	17.2 ± 0.3 a
0.25-Zn	<d.l.* e	<d.l.** e	0.18 ± 0.01 a	709 ± 39 a	<d.l.** d	4.2 ± 0.5 c
0.5-Zn	<d.l.* e	<d.l.** e	0.10 ± 0.01 c	508 ± 51 b	<d.l.** d	3.1 ± 0.3 cd
**2.5-Cd**						
No-Zn	1.21 ± 0.09 d	32 ± 2 c	0.10 ± 0.02 c	421 ± 19 cd	94 ± 4 c	11.3 ± 0.4 b
0.25-Zn	1.33 ± 0.06 d	27 ± 1 d	0.16 ± 0.01 ab	464 ± 25 bc	105 ± 8 c	4.3 ± 0.4 c
0.5-Zn	1.47 ± 0.01 d	33 ± 1 c	0.12 ± 0.02 c	473 ± 37 bc	103 ± 9 c	2.8 ± 0.5 cd
**5-Cd**						
No-Zn	2.93 ± 0.03 b	50 ± 2 a	0.12 ± 0.01 bc	351 ± 11 d	219 ± 9 a	11.4 ± 1.8 b
0.25-Zn	2.72 ± 0.03 c	48 ± 3 b	0.13 ± 0.01 bc	427 ± 18 cd	185 ± 11 b	4.2 ± 0.2 c
0.5-Zn	3.20 ± 0.12 a	52 ± 2 a	0.10 ± 0.02 c	374 ± 41 d	217 ± 17 a	2.3 ± 0.1 d

Means in a column with different letters were significantly different (P ≤ 0.05) according to the Fisher’s protected LSD test for the interaction soil Cd x foliar Zn.

* <l.d. = below detection limit of 0.002 mg kg^-1^ for soil DTPA-extractable soil Cd

** <l.d = below detection limit of 0.05 mg Cd kg^-1^ for shoot digests

**Table 3:** Root length and shoot phytate:Zn molar ratio in *L*. *rigidum*, and DTPA-extractable soil Cd and shoot DW in *T*. *subterraneum* as affected by the soil Cd treatment. Mean ± standard error.

	*L*. *rigidum*		*T*. *subterraneum*	
	Root length (cm plant^-1^)	Shoot phytate:Zn ratio	DTPA-extractable soil Cd (mg kg^-1^)	Shoot DW (g plant^-1^)
No-Cd	467 ± 36 a	10.6 ± 1.5 b	b.d.l.*	0.14 ± 0.02 a
2.5-Cd	420 ± 29 ab	11.6 ± 1.5 ab	1.3 ± 0.1 b	0.12 ± 0.01 b
5-Cd	373 ± 23 b	12.2 ± 1.7 a	3.1 ± 0.1 a	0.11 ± 0.01 b

Means in a column with different letters were significantly different (P ≤ 0.05) according to the Fisher’s protected LSD test for the soil Cd treatment.

* <l.d = below detection limit of 0.002 mg kg^-1^ for soil DTPA-extractable soil Cd

**Table 4:** DTPA-extractable soil Zn, root length, shoot dry weight (DW), and shoot Zn concentration and phytate:Zn molar ratio in *L*. *rigidum*, and DTPA-extractable soil Cd and Zn, average root diameter, shoot DW, and shoot Zn and protein concentration in *T*. *subterraneum* as affected by the foliar Zn treatment. Mean ± standard error.

*L*. *rigidum*						
	DTPA-extractable soil Zn (mg kg^-1^)	Root length (cm plant^-1^)	Shoot DW (g plant^-1^)	Shoot Zn concentration (mg kg^-1^)	Shoot phytate:Zn ratio	
No-Zn	0.21 ± 0.03 b	376 ± 25 b	0.34 ± 0.01 b	40 ± 1 c	16.7 ± 0.6a	
0.25-Zn	0.26 ± 0.03 a	480 ± 32 a	0.38 ± 0.01 a	67 ± 3 b	10.0 ± 0.5b	
0.5-Zn	0.28 ± 0.03 a	403 ± 27 b	0.35 ± 0.01 b	88 ± 7 a	7.7 ± 0.5c	
*T*. *subterraneum*						
	DTPA-extractable soil Cd (mg kg^-1^)	DTPA-extractable soil Zn (mg kg^-1^)	Average root diameter (mm)	Shoot DW (g plant^-1^)	Shoot Zn concentration (mg kg^-1^)	Protein concentration (g kg^-1^)
No-Zn	1.5 ± 0.05 a	0.27 ± 0.03 b	0.31 ± 0.04 a	0.11 ± 0.01 b	52 ± 5 c	391 ± 8 b
0.25-Zn	1.3 ± 0.05 b	0.36 ± 0.03 a	0.29 ± 0.05 ab	0.14 ± 0.01 a	154 ± 6 b	399 ± 9 ab
0.5-Zn	1.5 ± 0.08 a	0.38 ± 0.03 a	0.27 ± 0.08 b	0.11 ± 0.01 b	250 ± 16 a	413 ± 7 a

Means in a column with different letters were significantly different (P ≤ 0.05) according to the Fisher’s protected LSD test for the foliar Zn treatment.

DTPA-extractable soil Zn was influenced by foliar Zn application and plant species (Table 1). After plant growth, soil Zn concentration was significantly higher in the 0.25-Zn and 0.5-Zn compared with the no-Zn treatment. DTPA-extractable Zn was lower (by 13–18% depending on the Zn treatment) in soil in which *L*. *rigidum* had been grown compared with *T*. *subterraneum* (Table 4). Importantly, in all treatments, DTPA-extractable soil Zn was below the minimum required concentration of 0.5 mg kg^-1^ [32], suggesting low soil Zn availability and emphasising the importance of Zn biofortification to increase Zn content in the food chain.

### Effects of Cd and Zn treatments on root and shoot properties

A decrease in plant biomass is considered the first indication of Cd toxicity in plants grown on contaminated soils [33]. Nevertheless, plant species differ greatly in their tolerance and capacity to take up and transport Cd within the plant (eg. differences between *L*. *perenne* and *T*. *repens* [34] or four wetland species [7]). The toxicity can also differ depending on the growth stage [26]. In the present study, large differences were found between the studied species (Tables 2, 3 and 4). The growth of *L*. *rigidum* was generally not influenced by soil Cd treatment (root and shoot dry weights, Table 1), with only root length being decreased by about 84% at the highest soil Cd application compared with the 0-Cd and 2.5-Cd treatments (Table 3). On the other hand, in *T*. *subterraneum* Cd toxicity significantly decreased (Table 1) root length (on average by 68%, Table 2) and shoot dry weight (by about 79%, Table 3) compared with the non-contaminated control. Similarly to annual ryegrass (*L*. *rigidum*) (Table 3), an earlier study [35] showed the tolerance of perennial ryegrass (*L*. *perenne*) to different heavy metals and its capacity to accumulate metals in tissues. Regarding *T*. *subterraneum* (Tables 2 and 3), similar negative effects of Cd were also reported in a different clover species (*T*. *repens*) [36].

Although the growth of both species was positively influenced by the foliar application of 0.25% w/v ZnSO_4_·7H_2_O compared with the no-Zn treatment, such an improvement was significant in *L*. *rigidum* only regarding root length and shoot biomass (Tables 1 and 4), whereas in *T*. *subterraneum* there was improvement in root dry weight and length (Tables 1 and 2) and shoot dry weight (Tables 1 and 4). Moreover, this low Zn application significantly alleviated a Cd-induced loss in root dry weight in *T*. *subterraneum* (a similar, albeit non-significant, effect was noted for root length) (Tables 1 and 2). These findings are in line with the previous findings of Saifullah et al. [26] who demonstrated Zn foliar spray could be an effective treatment for ameliorating the adverse effects of Cd on wheat growth.

### Effects of Cd and Zn treatments on nutritional composition of pasture species

Shoot Cd concentration was significantly influenced by the interaction of soil Cd x foliar Zn in both *L*. *rigidum* and *T*. *subterraneum* (Table 1). Total shoot Cd concentrations ranged between 0.01 and 52 mg Cd kg^-1^ in *L*. *rigidum* and between 0.01 and 219 mg Cd kg^-1^ in *T*. *subterraneum* (Table 2), showing a large difference between the species in their capacity to take up and transport Cd within the plant. Compared with *L*. *rigidum*, *T*. *subterraneum* shoot Cd concentration was 3.3- and 4.1-fold higher in the 2.5-Cd and 5-Cd treatments, respectively. Also, based on the defined threshold (shoot Cd concentration >100 mg kg^-1^) for hyperaccumulation [37], in the present study *T*. *subterraneum* reached that level in the 2.5-Cd treatment and exceeded it by about 2-fold in the 5-Cd treatment. However, its shoot biomass decreased in the Cd treatments compared with the control, suggesting that the potential of *T*. *subterraneum* as a Cd hyperaccumulator in phytoremediation application is unclear at this stage; further testing of various genotypes as well as different agronomic practices is needed to ascertain the capacity of *T*. *subterraneum* to sustain growth at high shoot Cd concentrations. The application of 0.25% w/v ZnSO_4_·7H_2_O decreased total Cd concentration in shoots in the 2.5-Cd and 5-Cd treatments in *L*. *rigidum* and in the 5-Cd treatment in *T*. *subterraneum* compared with the No-Zn control (Tables 1 and 2). These results are in accordance with the recent studies on wheat (*Triticum aestivum* L.) [26,38] whereby a significant decrease in Cd concentration in various plant tissues (including shoots) occurred when foliar 0.3% w/v ZnSO_4_ was applied at the booting stage. Similar results were obtained with maize (*Zea mays* L.) as well, with an increase in grain yield due to Zn fertilization causing a decrease in Cd concentrations because of dilution [39]. Zinc is easily mobile in phloem, meaning that transport of foliarly-applied Zn down to roots and even exudation into the rhizosphere soil can occur [40]. This exuded Zn could be associated with a decline in Cd absorption by roots and translocation to shoots and grains [41]; in addition, it is possible that the interaction between root Zn and Cd uptake from soil would also occur due to allosteric regulation of Cd uptake from soil by the Zn-specific transporters.

Shoot Zn concentration in both species was significantly influenced by foliar Zn application in a dose-dependent manner (Table 1). Compared with the no-Zn control, the treatment with the low-dose of 0.25% w/v ZnSO_4_·7H_2_O increased shoot Zn concentration by 68 and 196% in *L*. *rigidum* and *T*. *subterraneum*, respectively (Table 4). Compared with the low-dose Zn application, a higher dose of 0.5% w/v ZnSO_4_·7H_2_O increased shoot Zn concentration by a further 31 and 62% in *L*. *rigidum* and *T*. *subterraneum*, respectively (Table 4). Taking into account that 30 and 50 mg Zn kg^-1^ are the target levels for pastures to achieve a sufficient Zn status in sheep and cattle, respectively [42], shoot Zn concentration in this study was adequate even in the No-Zn treatment. However, a higher Zn concentration in pastures [up to 100 mg Zn kg^-1^ [43] is related to beneficial health effects in animals, with toxicity occurring around 500 mg Zn kg^-1^ [44]. In all of the Zn treatments, *T*. *subterraneum* had a higher shoot Zn concentration than *L*. *rigidum* (1.3, 2.3 and 2.8 times more in the No-Zn, 0.25-Zn and 0.5-Zn treatments, respectively, Table 4). These results on the strong positive effects of Zn application on increasing shoot Zn concentrations in pasture plants were consistent with studies on using Zn fertilization to increase Zn concentration in grains of wheat [26] and field pea [8].

Bioavailability of metals in the animal/human digestive systems may be assessed via their molar ratios with phytate. Molar ratios of phytate:Zn >15 [45] represent reduced Zn bioavailability. In the present study, shoot phytate:Zn ratio was influenced by soil Cd and foliar Zn treatments in both plant species, and also by their interaction in *T*. *subterraneum* (Table 1). In *L*. *rigidum*, the phytate:Zn ratio ranged from 7.7 to 16.7; it was increased by the soil Cd treatment (Table 3) and decreased by the increased Zn dose (Table 4). In *T*. *subterraneum*, the phytate:Zn ratios ranged between 2.3 and 17.2, with the lowest ratio (best bioavailability) at the highest foliar Zn dose regardless of the soil Cd treatment (Table 2).

Protein concentrations in shoots were, on average, 215 and 401 g kg^-1^ in *L*. *rigidum* and *T*. *subterraneum*, respectively. Shoot protein concentration was significantly influenced only in *T*. *subterraneum* and only by the Zn treatment (Table 1). In contrast, a negative effect of Cd on shoot protein concentration was found by Luo et al. [46] in *L*. *perenne*. It should be borne in mind that the relatively high shoot protein concentration in legume *T*. *subterraneum* may result in an increased capacity to accumulate minerals such as Zn and Mg [47].

## Conclusions

Foliar application of 0.25% w/v ZnSO_4_·7H_2_O decreased shoot Cd concentration in *L*. *rigidum* and *T*. *subterraneum* grown in Cd-contaminated soil and ameliorated the adverse effects of Cd exposure on root growth, mainly in *T*. *subterraneum*. Ryegrass *L*. *rigidum* had higher tolerance to Cd stress (in terms of maintaining root growth and relatively low shoot Cd concentration) than *T*. *subterraneum*. Further pot and field experiments with Cd-contaminated soils are needed to evaluate Zn-Cd interactions in different pasture species to clarify the underlying mechanisms as well as to devise practical approaches to minimising Cd-related growth and nutrition problems.

## Supporting information

S1 Table Mean ± standard error of DTPA-extractable soil Cd and Zn, root DW, root length, average root diameter, shoot DW, shoot Cd and Zn concentrations, phytate:Zn molar ratio and shoot protein concentration in *L*. *rigidum*. * Detection limit < 0.002 mg kg^-1^ for soil DTPA-extractable soil Cd. ** Detection limit < 0.05 mg kg^-1^ for digested plants.
(PPTX)

S2 Table Mean ± standard error of DTPA-extractable soil Cd and Zn, root DW, root length, average root diameter, shoot DW, shoot Cd and Zn concentrations, phytate:Zn molar ratio and shoot protein concentration in *T*. *subterraneum*. * Detection limit < 0.002 mg kg^-1^ for soil DTPA-extractable soil Cd. ** Detection limit < 0.05 mg kg^-1^ for digested plants.
(PPTX)
