# Supplementary material for: Foliar zinc biofortification effects in Lolium rigidum and Trifolium subterraneum grown in cadmium-contaminated soil
Source: PLoS One. 2017 Sep 26;12(9):e0185395. doi: 10.1371/journal.pone.0185395 (PMC5614633; doi:10.1371/journal.pone.0185395)
Supplement: S1 Table — * Detection limit < 0.002 mg kg-1 for soil DTPA-extractable soil Cd. ** Detection limit < 0.05 mg kg-1 for digested plants. (PPTX) [file pone.0185395.s001.pptx]

## Slide 1
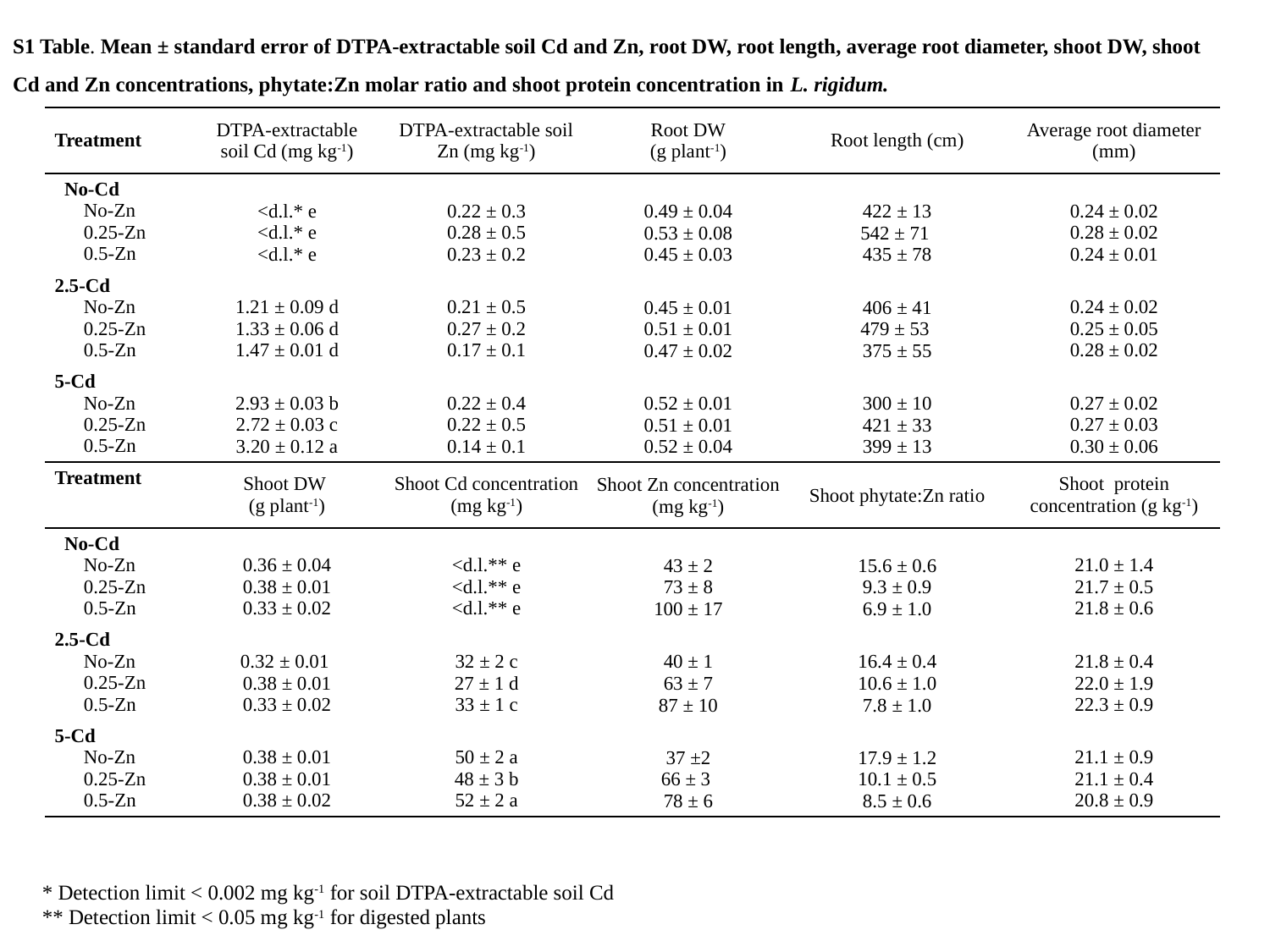

S1 Table. Mean ± standard error of DTPA-extractable soil Cd and Zn, root DW, root length, average root diameter, shoot DW, shoot Cd and Zn concentrations, phytate:Zn molar ratio and shoot protein concentration in L. rigidum.
| Treatment | DTPA-extractable soil Cd (mg kg-1) | DTPA-extractable soil Zn (mg kg-1) | Root DW (g plant-1) | Root length (cm) | Average root diameter (mm) |
| --- | --- | --- | --- | --- | --- |
| No-Cd No-Zn 0.25-Zn 0.5-Zn | <d.l.\* e <d.l.\* e <d.l.\* e | 0.22 ± 0.3 0.28 ± 0.5 0.23 ± 0.2 | 0.49 ± 0.04 0.53 ± 0.08 0.45 ± 0.03 | 422 ± 13 542 ± 71 435 ± 78 | 0.24 ± 0.02 0.28 ± 0.02 0.24 ± 0.01 |
| 2.5-Cd No-Zn 0.25-Zn 0.5-Zn | 1.21 ± 0.09 d 1.33 ± 0.06 d 1.47 ± 0.01 d | 0.21 ± 0.5 0.27 ± 0.2 0.17 ± 0.1 | 0.45 ± 0.01 0.51 ± 0.01 0.47 ± 0.02 | 406 ± 41 479 ± 53 375 ± 55 | 0.24 ± 0.02 0.25 ± 0.05 0.28 ± 0.02 |
| 5-Cd No-Zn 0.25-Zn 0.5-Zn | 2.93 ± 0.03 b 2.72 ± 0.03 c 3.20 ± 0.12 a | 0.22 ± 0.4 0.22 ± 0.5 0.14 ± 0.1 | 0.52 ± 0.01 0.51 ± 0.01 0.52 ± 0.04 | 300 ± 10 421 ± 33 399 ± 13 | 0.27 ± 0.02 0.27 ± 0.03 0.30 ± 0.06 |
| Treatment | Shoot DW (g plant-1) | Shoot Cd concentration (mg kg-1) | Shoot Zn concentration (mg kg-1) | Shoot phytate:Zn ratio | Shoot protein concentration (g kg-1) |
| No-Cd No-Zn 0.25-Zn 0.5-Zn | 0.36 ± 0.04 0.38 ± 0.01 0.33 ± 0.02 | <d.l.\*\* e <d.l.\*\* e <d.l.\*\* e | 43 ± 2 73 ± 8 100 ± 17 | 15.6 ± 0.6 9.3 ± 0.9 6.9 ± 1.0 | 21.0 ± 1.4 21.7 ± 0.5 21.8 ± 0.6 |
| 2.5-Cd No-Zn 0.25-Zn 0.5-Zn | 0.32 ± 0.01 0.38 ± 0.01 0.33 ± 0.02 | 32 ± 2 c 27 ± 1 d 33 ± 1 c | 40 ± 1 63 ± 7 87 ± 10 | 16.4 ± 0.4 10.6 ± 1.0 7.8 ± 1.0 | 21.8 ± 0.4 22.0 ± 1.9 22.3 ± 0.9 |
| 5-Cd No-Zn 0.25-Zn 0.5-Zn | 0.38 ± 0.01 0.38 ± 0.01 0.38 ± 0.02 | 50 ± 2 a 48 ± 3 b 52 ± 2 a | 37 ±2 66 ± 3 78 ± 6 | 17.9 ± 1.2 10.1 ± 0.5 8.5 ± 0.6 | 21.1 ± 0.9 21.1 ± 0.4 20.8 ± 0.9 |
* Detection limit < 0.002 mg kg-1 for soil DTPA-extractable soil Cd
** Detection limit < 0.05 mg kg-1 for digested plants
