# Supplementary material for: Foliar zinc biofortification effects in Lolium rigidum and Trifolium subterraneum grown in cadmium-contaminated soil
Source: PLoS One. 2017 Sep 26;12(9):e0185395. doi: 10.1371/journal.pone.0185395 (PMC5614633; doi:10.1371/journal.pone.0185395)
Supplement: S2 Table — * Detection limit < 0.002 mg kg-1 for soil DTPA-extractable soil Cd. ** Detection limit < 0.05 mg kg-1 for digested plants. (PPTX) [file pone.0185395.s002.pptx]

## Slide 1
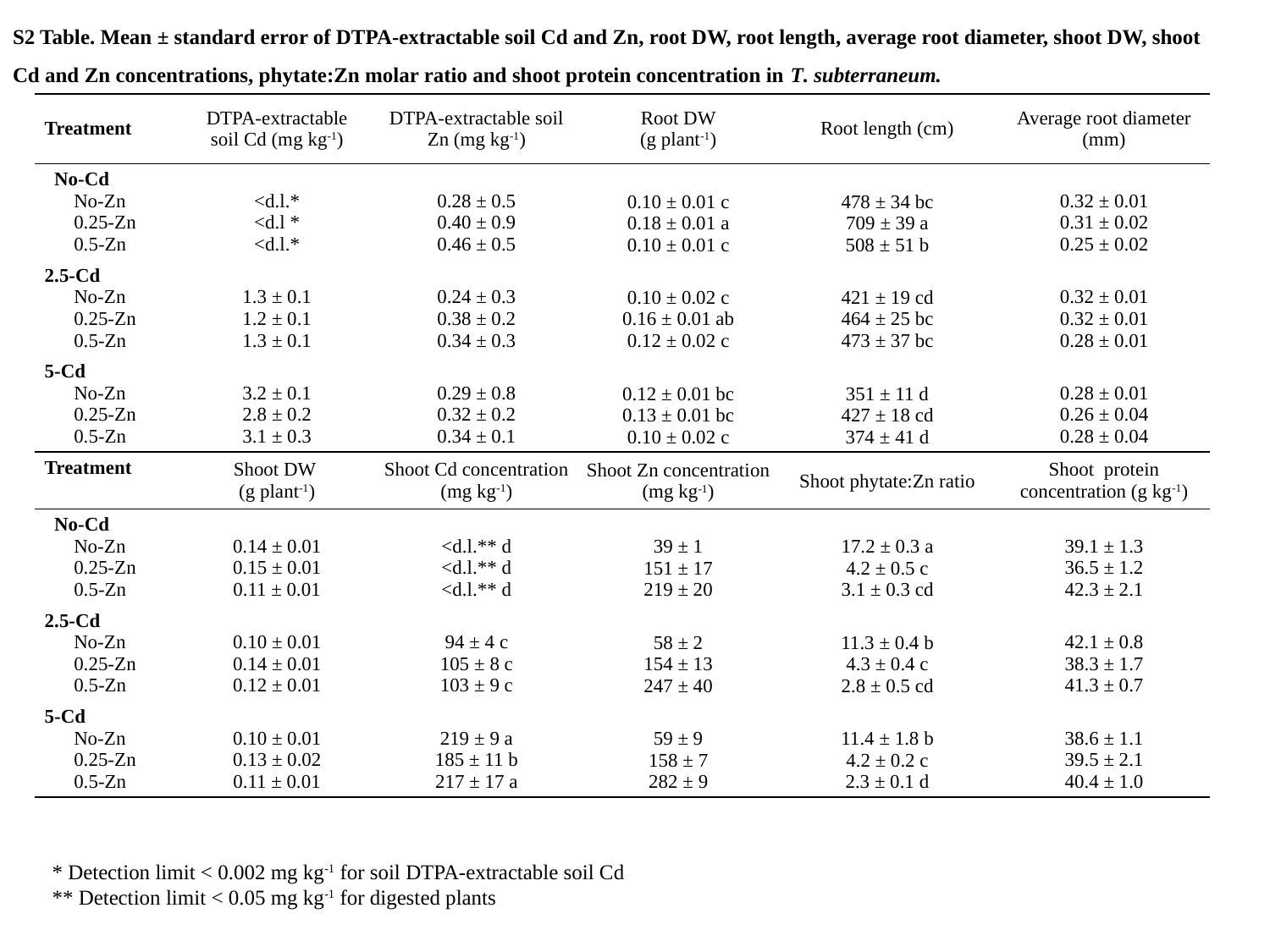

S2 Table. Mean ± standard error of DTPA-extractable soil Cd and Zn, root DW, root length, average root diameter, shoot DW, shoot Cd and Zn concentrations, phytate:Zn molar ratio and shoot protein concentration in T. subterraneum.
| Treatment | DTPA-extractable soil Cd (mg kg-1) | DTPA-extractable soil Zn (mg kg-1) | Root DW (g plant-1) | Root length (cm) | Average root diameter (mm) |
| --- | --- | --- | --- | --- | --- |
| No-Cd No-Zn 0.25-Zn 0.5-Zn | <d.l.\* <d.l \* <d.l.\* | 0.28 ± 0.5 0.40 ± 0.9 0.46 ± 0.5 | 0.10 ± 0.01 c 0.18 ± 0.01 a 0.10 ± 0.01 c | 478 ± 34 bc 709 ± 39 a 508 ± 51 b | 0.32 ± 0.01 0.31 ± 0.02 0.25 ± 0.02 |
| 2.5-Cd No-Zn 0.25-Zn 0.5-Zn | 1.3 ± 0.1 1.2 ± 0.1 1.3 ± 0.1 | 0.24 ± 0.3 0.38 ± 0.2 0.34 ± 0.3 | 0.10 ± 0.02 c 0.16 ± 0.01 ab 0.12 ± 0.02 c | 421 ± 19 cd 464 ± 25 bc 473 ± 37 bc | 0.32 ± 0.01 0.32 ± 0.01 0.28 ± 0.01 |
| 5-Cd No-Zn 0.25-Zn 0.5-Zn | 3.2 ± 0.1 2.8 ± 0.2 3.1 ± 0.3 | 0.29 ± 0.8 0.32 ± 0.2 0.34 ± 0.1 | 0.12 ± 0.01 bc 0.13 ± 0.01 bc 0.10 ± 0.02 c | 351 ± 11 d 427 ± 18 cd 374 ± 41 d | 0.28 ± 0.01 0.26 ± 0.04 0.28 ± 0.04 |
| Treatment | Shoot DW (g plant-1) | Shoot Cd concentration (mg kg-1) | Shoot Zn concentration (mg kg-1) | Shoot phytate:Zn ratio | Shoot protein concentration (g kg-1) |
| No-Cd No-Zn 0.25-Zn 0.5-Zn | 0.14 ± 0.01 0.15 ± 0.01 0.11 ± 0.01 | <d.l.\*\* d <d.l.\*\* d <d.l.\*\* d | 39 ± 1 151 ± 17 219 ± 20 | 17.2 ± 0.3 a 4.2 ± 0.5 c 3.1 ± 0.3 cd | 39.1 ± 1.3 36.5 ± 1.2 42.3 ± 2.1 |
| 2.5-Cd No-Zn 0.25-Zn 0.5-Zn | 0.10 ± 0.01 0.14 ± 0.01 0.12 ± 0.01 | 94 ± 4 c 105 ± 8 c 103 ± 9 c | 58 ± 2 154 ± 13 247 ± 40 | 11.3 ± 0.4 b 4.3 ± 0.4 c 2.8 ± 0.5 cd | 42.1 ± 0.8 38.3 ± 1.7 41.3 ± 0.7 |
| 5-Cd No-Zn 0.25-Zn 0.5-Zn | 0.10 ± 0.01 0.13 ± 0.02 0.11 ± 0.01 | 219 ± 9 a 185 ± 11 b 217 ± 17 a | 59 ± 9 158 ± 7 282 ± 9 | 11.4 ± 1.8 b 4.2 ± 0.2 c 2.3 ± 0.1 d | 38.6 ± 1.1 39.5 ± 2.1 40.4 ± 1.0 |
* Detection limit < 0.002 mg kg-1 for soil DTPA-extractable soil Cd
** Detection limit < 0.05 mg kg-1 for digested plants
